# Association between family income to poverty ratio and severe headache/migraine in the American adults: data from NHANES 1999–2004

**DOI:** 10.3389/fneur.2024.1427277

**Published:** 2024-11-18

**Authors:** Lingling Sun, Rongjian Zhao, Xuemei You, Junpeng Meng, Lin Meng, Haili Di

**Affiliations:** Department of Neurology, Northwest University First Hospital, Xi’an, China

**Keywords:** severe headache/migraine, family income to poverty ratio, NHANES, cross-sectional study, adult

## Abstract

**Background:**

The relationship between family income to poverty ratio (PIR) and severe headache/migraine remains unclear.

**Methods:**

Data for this cross-sectional study were obtained from NHANES 1999–2004. PIR was the exposure variable, and severe headache/migraine was the dependent variable. We performed univariate analyses of severe headache/migraine, PIR, and other covariates. The association between PIR and severe headache/migraine was tested using multiple regression models. Furthermore, interaction tests and stratified analyses assessed the relationship between PIR and severe headache/migraine across subgroups.

**Results:**

There were a total of 8,800 participants: 4,833 (54.92%) males and 3,967 (45.08%) females, 1,714 (19.48%) with severe headache/migraine and 7,086 (80.52%) without severe headache/migraine. After adjustment for all variables, PIR negatively correlated with severe headache/migraine OR = 0.86 95% CI (0.83, 0.90) *p* < 0.0001. The variable PIR was categorized as the low-income (PIR < 1), the middle-income (1 ≤ PIR < 4), and the high-income (PIR ≥ 4). Notably, there was a significant difference in trend for the high-income group (PIR ≥ 4) compared to the control low-income group (PIR < 1) (all *P* for interaction<0.05). Dose–response correlations were also analyzed using smoothed curve fitting, revealing a negative correlation between PIR and severe headache/migraine (*p* < 0.0001). Subgroup analysis results indicated that the negative association between PIR and severe headache/migraine was more pronounced in the following populations: males (OR = 0.84 95% CI (0.79, 0.90), <60 years old [Age < 45 group OR = 0.81 95% CI (0.76, 0.85)], Age 45–60 group OR = 0.86 95% CI (0.79, 0.93), and those with education levels ≥high school [High School OR = 0.87 95% CI (0.81, 0.95), >High School OR = 0.82 95% CI (0.78, 0.87)].

**Conclusion:**

There is a negative correlation between PIR and the incidence of severe headaches/ migraine in Americans aged 20 years or older. This study has implications for the comprehensive management of patients with severe headache/migraine.

## 1 Introduction

Migraine is a common neurological disorder. A Global Burden of Disease Survey report indicates that migraines are the second most common condition worldwide and the top disease among young women (1). Up to 15% of American adults suffer from migraines annually, with migraine affecting about 1 billion people globally. Before puberty, boys are more likely than girls to have migraines. However, after puberty, females are three times more likely to have migraines than males, with a peak incidence in the 40s and 50s (2). Notably, migraine is a complex neurologic disorder that is related to several aspects of the patient (psychological, personal, and economic) (3). Therefore, individuals’ lives are significantly impacted by migraine, placing a significant strain on society.

The Family Income to Poverty Ratio (PIR) is a reliable indicator of income inequality. In this model, higher values denote better household economic circumstances. Importantly, PIR and the incidence of disease are highly correlated. Lower socioeconomic level is strongly associated with cardiovascular disease morbidity and mortality, according to prior study results (4–7). In contrast, the likelihood of infections, mental illnesses, asthma, anemia, and 10-year mortality in children is inversely correlated with family wealth (8). Low family incomes may also be a cause of risk for childhood asthma, according to a Japanese study (9). From their investigation, Zhao Y et al. demonstrated that a lower PIR was linked to a higher incidence of HPV infections (10). Another study on American women revealed a connection between PIR and abdominal fat (11). However, there has not been any research done on the connection between severe headaches/migraine and PIR.

To explore the connection between PIR and severe headache/migraine, we used data from the NHANES 1999–2004 to perform this cross-sectional analysis.

## 2 Methods

### 2.1 Study populations

The National Health and Nutrition Examination Survey (NHANES) is an American population-based cross-sectional survey database designed to assess the health and nutritional status of adults and children in the United States. The National Center for Health Statistics (NCHS) Research Ethics Review Board approved the study procedure. Before administering the survey, written consent was obtained from all participants. The results of NHANES can be used to investigate the prevalence of major diseases and risk factors for disease. Detailed information about NHANES is available at https://www.cdc.gov/nchs/NHANES/.

The statistical data for this study came from NHANES (1999–2004), which contains data from 3 cycles. NHANES 1999–2004 was chosen because we could only screen the population for severe headaches/migraines in these cycles in our study.

**Figure 1:**
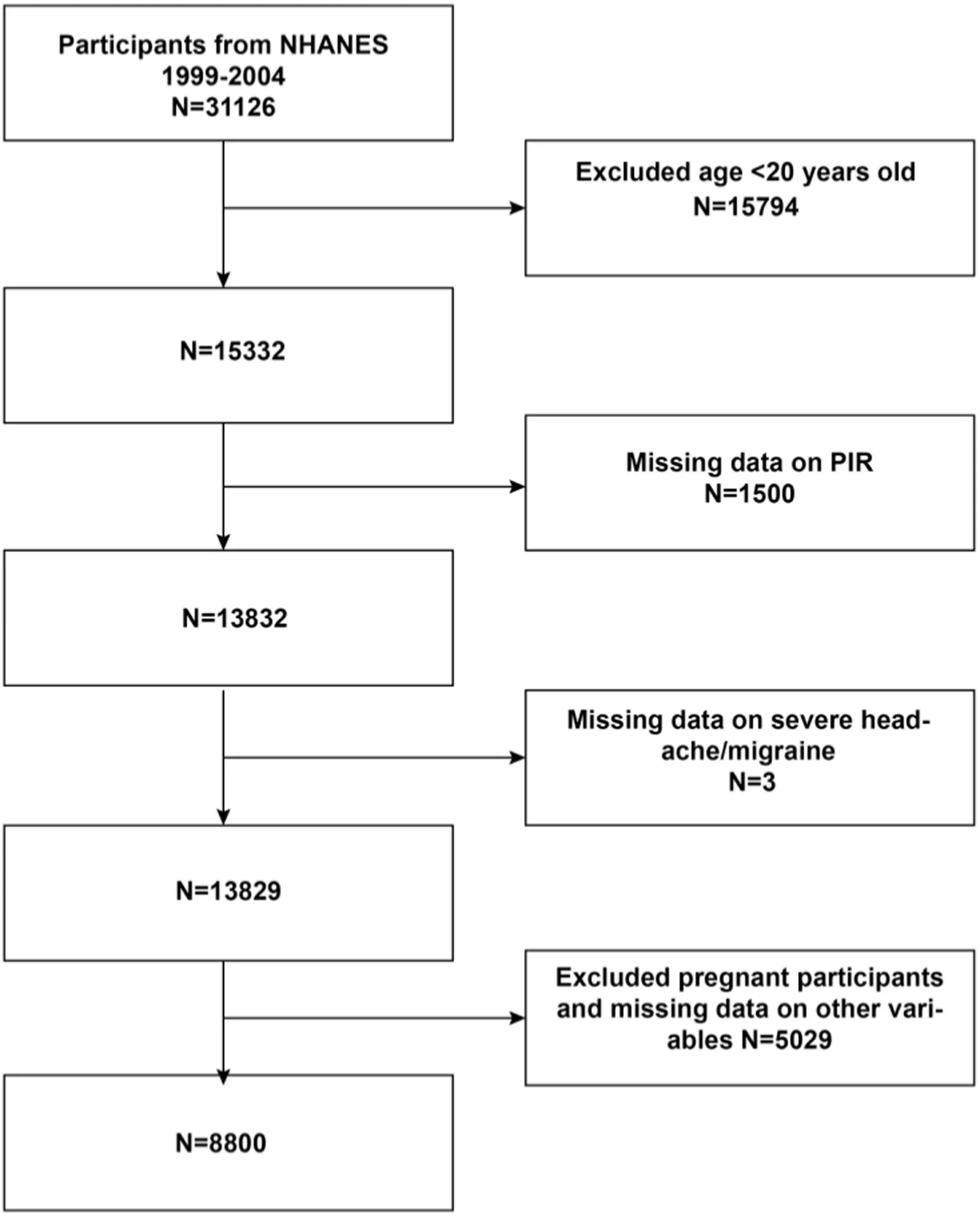
Flowchat of participant selection from NHANES 1999-2004. PIR Family Income to Poverty Ratio.

Initially, the study included 31,126 participants. The exclusion criteria were age less than 20 years (*n* = 15,794), those with missing data on PIR (*n* = 1,500), those with missing data on severe headache/migraine (*n* = 3), those with missing data on other variables, and those who were pregnant (*n* = 5,029). Finally, 8,800 eligible participants were included in the present study (the detailed process is shown in Figure 1).

### 2.2 Study variables

The exposure variable was PIR, and the outcome variable was severe headache/migraine. PIR was determined using the Consumer Price Index (CPI) based on household income and federally recognized poverty level. A PIR of less than 1 (100 percent of the federal poverty level) indicates that a family’s income is below the poverty line and is a low-income family (12). And PIR of 4 and above (400 percent or more of the federal poverty level) for high-income households (13). We then categorized these into three classes: the low-income (PIR < 1), the middle-income (1 ≤ PIR < 4), and the high-income (PIR ≥ 4). Multiple studies have used the same categorization (10, 13–17). Information on severe headaches/migraines had been obtained from questionnaires, which is the only way migraineurs can be screened. MPQ090 - During the past 3 months, did you have severe headaches or migraines? Those who answered “yes” were recognized as having severe headaches/migraines; those who answered “no” were identified as having non-severe headaches/migraines. Notably, this method has been used in several cross-sectional studies to screen patients with severe headaches or migraines (18–21).

The covariates in this study were age, gender, race, education (<high school, high school, >high school), marital status, hypertension, diabetes, stroke, coronary heart disease, smoking, alcohol drinking, Body Mass Index (BMI), and coffee intake. We obtained age, gender, race, marital status, and education from the Demographics Data, BMI from the Examination Data, and coffee intake from the Dietary Data. Drinking information was obtained by asking the question - Was there ever a time or times in your life when you drank five or more drinks of any alcoholic beverage almost every day? Answering “yes” was assigned to the drinking group, and answering “no” was assigned to the non-drinking group. Smoking was obtained by asking the question - Have you smoked at least 100 cigarettes in your entire life? Answering “yes” was assigned to the smoking group, and answering “no” was assigned to the nonsmoking group. Additionally, information on hypertension, diabetes, stroke, and coronary heart disease was obtained through questionnaire questions. Questions include: Have you ever been told by a doctor that you have hypertension? Have you ever been told by a doctor that you have diabetes? Have you ever been told by a doctor that you have a stroke? Have you ever been told by a doctor that you have coronary heart disease? We grouped participants based on responses. You can find explanations for the above variables on the official page of the NHANES website.

### 2.3 Statistical analysis

We used R (version 4.1.3) and EmpowerStats (version 2.0) for the statistical data analysis. Continuous variables are presented as mean ± SD and were compared across the three PIR groups (PIR < 1, 1 ≤ PIR < 4, PIR ≥ 4) using the Kruskal–Wallis rank-sum test; categorical variables are presented as *n* (%) and were compared using the chi-square test. We also performed univariate analyses between outcome variables, exposure variables, and covariates, and the association between PIR and severe headache/migraine was tested using multiple regression models. Furthermore, dose–response correlations were analyzed using smoothed curve fitting. Moreover, interaction tests and stratified analyses assessed the association between PIR and severe headache/migraine across subgroups. The difference was considered statistically significant at *p* < 0.05.

## 3 Results

### 3.1 Baseline characteristics of participants

Baseline characteristics of the population (Table 1) showed a total of 8,800 participants, 4,833 (54.92%) males and 3,967 (45.08%) females. There were 1,714 participants (19.48%) with severe headache/migraine and 7,086 (80.52%) without severe headache/migraine. The mean age was 50.22 ± 18.12, and the mean PIR was 2.75 ± 1.60. There were 1,385 (15.74%) participants with low-income (PIR < 1), 4,778 (54.30%) with middle-income (1 ≤ PIR < 4), and 2,637 (29.96%) with high-income (PIR ≥ 4). Notably, the low-income individuals (PIR < 1) were younger, more likely to have less than a high school education, had lower coffee intake, and had a higher prevalence of severe headaches/migraines compared to the high-income individuals (PIR ≥ 4).

**Table 1:** Basic characteristics of participants.

Variables	Low income (PIR < 1 *n* = 1,385)	Middle income (1 ≤ PIR < 4 *n* = 4,778)	High income (PIR ≥ 4 *n* = 2,637)	*P*-value
Age(years)	47.25 ± 18.69	51.12 ± 19.08	50.16 ± 15.71	<0.001
Gender, *n* (%)				0.361
Male	740 (53.43%)	2,622 (54.88%)	1,471 (55.78%)	
Female	645 (46.57%)	2,156 (45.12%)	1,166 (44.22%)	
Race, *n* (%)				<0.001
Mexican American	463 (33.43%)	1,100 (23.02%)	281 (10.66%)	
Other Hispanic	90 (6.50%)	229 (4.79%)	49 (1.86%)	
Non-Hispanic White	435 (31.41%)	2,451 (51.30%)	1,888 (71.60%)	
Non-Hispanic Black	347 (25.05%)	882 (18.46%)	332 (12.59%)	
Other race	50 (3.61%)	116 (2.43%)	87 (3.30%)	
Education, *n* (%)				<0.001
<High school	787 (56.82%)	1,560 (32.65%)	211 (8.00%)	
High school	268 (19.35%)	1,332 (27.88%)	500 (18.96%)	
>High school	330 (23.83%)	1,886 (39.47%)	1,926 (73.04%)	
Marital status, *n* (%)							<0.001
Married	545 (39.35%)	2,636 (55.17%)	1,881 (71.33%)	
Widowed	130 (9.39%)	506 (10.59%)	125 (4.74%)	
Divorced	185 (13.36%)	483 (10.11%)	195 (7.39%)	
Separated	99 (7.15%)	141 (2.95%)	40 (1.52%)	
Never married	297 (21.44%)	735 (15.38%)	296 (11.22%)	
Living with partner	129 (9.31%)	277 (5.80%)	100 (3.79%)	
Diabetes, *n* (%)				<0.001
Yes	176 (12.71%)	498 (10.42%)	155 (5.88%)	
No	1,194 (86.21%)	4,203 (87.97%)	2,455 (93.10%)	
Borderline	15 (1.08%)	77 (1.61%)	27 (1.02%)	
Hypertension, *n* (%)				0.011
Yes	433 (31.26%)	1,590 (33.28%)	790 (29.96%)	
No	952 (68.74%)	3,188 (66.72%)	1847 (70.04%)	
Stroke, *n* (%)				<0.001
Yes	57 (4.12%)	169 (3.54%)	50 (1.90%)	
No	1,328 (95.88%)	4,609 (96.46%)	2,587 (98.10%)	
Coronary heart disease, *n* (%)				0.782
Yes	62 (4.48%)	232 (4.86%)	131 (4.97%)	
No	1,323 (95.52%)	4,546 (95.14%)	2,506 (95.03%)	
Smoke status, *n* (%)				<0.001
Yes	891 (64.33%)	2,710 (56.72%)	1,290 (48.92%)	
No	494 (35.67%)	2068 (43.28%)	1,347 (51.08%)	
Drink status, *n* (%)				<0.001
Yes	320 (23.10%)	902 (18.88%)	295 (11.19%)	
No	1,065 (76.90%)	3,876 (81.12%)	2,342 (88.81%)	
BMI (kg/m2)	32.70 ± 8.11	32.72 ± 7.94	32.40 ± 8.20	0.135
Caffeine intake (mg/d)	73.00 (4.00, 192.00)	112.00 (26.00, 246.02)	146.77 (49.70, 289.00)	
Migraines, *n* (%)				<0.001
Yes	376 (27.15%)	970 (20.30%)	368 (13.96%)	
No	1,009 (72.85%)	3,808 (79.70%)	2,269 (86.04%)	

### 3.2 Results of single factor analysis

Results from the single-factor analysis (Table 2) suggested that gender, race, education, marital status, age, and coronary heart disease may be associated with severe headache/migraine.

**Table 2:** The association of variables and severe headache/migraine risk.

Variables	OR (95% CI) *p*- value	Variables	OR (95% CI) *p*- value
Gender		Diabetes	
Male	1 (reference)	Yes	1 (reference)
Female	2.17 (1.95, 2.42) <0.0001	No	1.14 (0.95, 1.38) 0.1586
Race		Borderline	1.24 (0.77, 2.00) 0.3682
Mexican American	1 (reference)	Hypertension	
Other Hispanic	1.19 (0.92,1.56) 0.1871	Yes	1(reference)
Non-Hispanic White	0.83 (0.72,0.95) 0.0057	No	1.03 (0.92, 1.15) 0.6486
Non-Hispanic Black	1.02 (0.87,1.21) 0.7862	Stroke	
Other race	1.13 (0.83,1.55) 0.4426	Yes	1 (reference)
Education		No	0.85 (0.63, 1.13) 0.2639
<High School	1 (reference)	Coronary heart disease	
High school	0.98 (0.85, 1.13) 0.8215	Yes	1 (reference)
>High school	0.82 (0.72, 0.92) 0.0014	No	1.59 (1.20, 2.12) 0.0013
Marital status		Smoke status	
Married	1 (reference)	Yes	1 (reference)
Widowed	0.64 (0.51, 0.81) 0.0002	No	1.03 (0.92, 1.14) 0.6400
Divorced	1.34 (1.12, 1.59) 0.0013	Drink status	
Separated	1.93 (1.48, 2.53) <0.0001	Yes	1 (reference)
Never married	1.36 (1.17, 1.57) <0.0001	No	0.92 (0.80, 1.06) 0.2389
Living with partner	2.08 (1.70, 2.55) <0.0001	Age (years)	0.98 (0.97, 0.98) <0.0001
Caffeineintake (mg/d)	1.00 (1.00, 1.00) 0.0577	BMI (kg/m2)	1.00 (1.00, 1.01) 0.1914
PIR	0.83 (0.80, 0.86) <0.0001		

### 3.3 Correlation of PIR with severe headache/migraine

**Figure 2:**
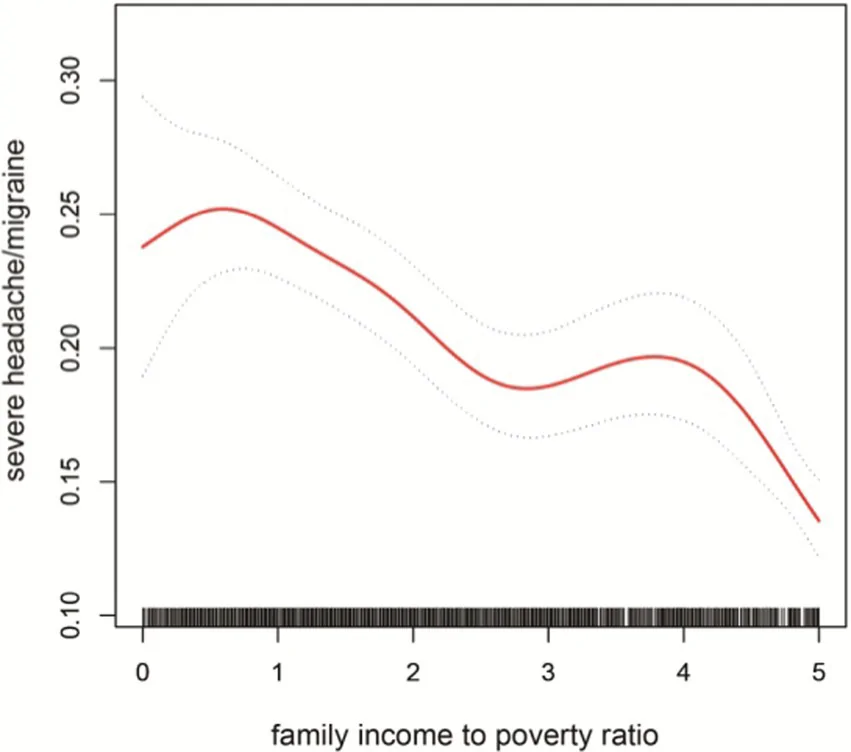
The solid red line represent the smooth curve fit between variables. Blue bands represent the 95% of confidence inter- val from the fit.

Multiple logistic regression was used to analyze the association between PIR and severe headache/migraine. The results are shown in Table 3. Model I did not adjust for any variables; model II adjusted for the results of single-factor analyses correlated with severe headache/migraine, including covariates (gender, race, education, marital status, age, coronary heart disease). Model III adjusted for all covariates (age, gender, race, education, marital status, hypertension, diabetes, stroke, coronary heart disease, smoking, drinking, BMI, coffee intake). Model I OR = 0.83 95% CI (0.80, 0.86) *p* < 0.0001, Model II OR = 0.85 95% CI (0.82, 0.89) *p* < 0.0001, Model III OR = 0.86 95% CI (0.83, 0.90) *p* < 0.0001. Thus, all three models suggested that PIR and the risk of severe headache/migraine showed a negative correlation, implying that the risk of severe headache/migraine will decrease as PIR rises. After triple categorizing the variable PIR (PIR < 1, 1 ≤ PIR < 4, PIR ≥ 4), there was a significant difference in trend for the high-income group (PIR ≥ 4) compared to the low-income group (PIR < 1). One study grouped PIR like this: low-income (PIR < 1.3), middle-income (PIR 1.3–3.5,) and high-income (PIR > 3.5) (22). We also analyzed the correlation between PIR and severe headache/migraine using multivariate logistic regression after triple categorizing the variable PIR (PIR < 1.3, PIR1.3–3.5, PIR > 3.5). The results are shown in Table 4. Similarly, it was found that the risk of severe headache/migraine was reduced with the increase of PIR, and both of them are somewhat negatively correlated. Furthermore, there was a significant difference in trend for the high-income group (PIR > 3.5) compared to the low-income group (PIR < 1.3). Moreover, we fitted a smooth curve to the nonlinear connection between PIR and severe headache/migraine. The outcomes showed a nonlinear negative association between the two variables (*p* < 0.0001; Figure 2).

**Table 3:** The association between family PIR and migraine.

Exposure	Model I OR(95% CI) *P*	Model II OR(95% CI) *P*	Model III OR(95% CI) *P*
PIR	0.83 (0.80, 0.86) <0.0001	0.85 (0.82, 0.89) <0.0001	0.86 (0.83, 0.90) <0.0001
PIR classification			
Low income (PIR < 1)	Reference	Reference	Reference
Middle income (1 ≤ PIR < 4)	0.68 (0.60, 0.78) <0.0001	0.78 (0.68, 0.91) <0.0001	0.80 (0.69, 0.92) 0.0028
High income (PIR ≥ 4)	0.44 (0.37, 0.51) <0.0001	0.52 (0.43, 0.63) <0.0001	0.54 (0.45, 0.65) <0.0001
*P* for trend	<0.001	<0.001	<0.001

Model I, None covariates were adjusted; Model II, Age, race, marital status, education and coronary heart disease were adjusted; Model III, Age, race, marital status, education, coronary heart disease, drink and smoke status, Caffeine intake, Hypertension, diabetes, stroke, and BMI (body mass index) were adjusted. PIR is the ratio of family income to poverty.

**Table 4:** The association between PIR and migraine.

Exposure	Model I OR(95% CI) *p*	Model II OR(95% CI) *p*	Model III OR(95% CI) *p*
PIR	0.83 (0.80, 0.86) <0.0001	0.85 (0.81, 0.88) <0.0001	0.86 (0.83, 0.90) <0.0001
PIR classification			
Low income(PIR < 1.3)	Reference	Reference	Reference
Middle income(PIR1.3–3.5)	0.72(0.63, 0.82) <0.0001	0.79 (0.69, 0.90) 0.0004	0.81 (0.70, 0.92) 0.0020
High income(PIR ≥ 3.5)	0.51 (0.45, 0.59) <0.0001	0.56 (0.48, 0.66) <0.0001	0.60 (0.51, 0.70) <0.0001
*p* for trend	<0.001	<0.001	<0.001

Model I: None covariates were adjusted; Model II: Age, race, marital status, education and coronary heart disease were adjusted; Model III: Age, race, marital status, education, coronary heart disease, drink status, smoke status, Caffeine intake, Hypertension, diabetes, stroke, and BMI(body mass index)were adjusted. PIR is the ratio of family income to poverty.

### 3.4 Subgroup analysis

**Figure 3:**
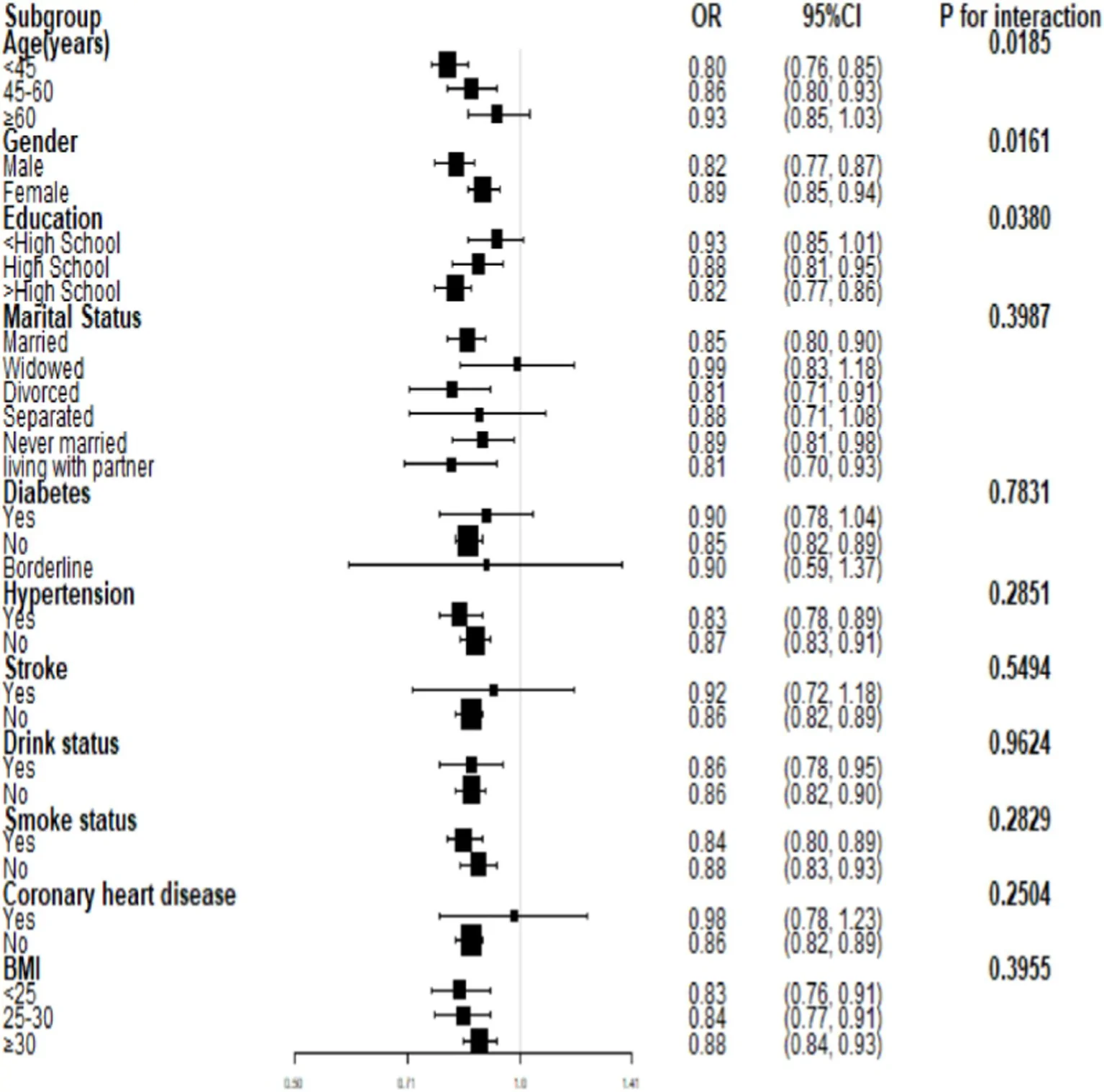
Subgroup analysis for the association between PIR and severe headache/migraine.

Interaction tests (Figure 3) were performed after stratifying by age, gender, marital status, education, smoking, alcohol drinking, hypertension, diabetes, stroke, coronary heart disease, and BMI. The results suggested that the negative association between PIR and severe headache/migraine was not significant among the subgroups of marital status, hypertension, diabetes, stroke, smoking, alcohol drinking, coronary heart disease, and BMI. In contrast, differences were noted among the subgroups of age, gender, and education. In the gender subgroups of Figure 3, each increase in PIR was associated with an 18% reduction in the risk of severe headache/migraine in men, and each increase in PIR was associated with an 11% reduction in the risk of severe headache/migraine in women. In both men and women, PIR was negatively associated with severe headache/migraine, with the negative association being more pronounced in men (*p* = 0.016). We then stratified the analysis (Table 5) among the subgroups of age, gender, and education. Combining the results of the interaction test and stratified analyses, we found that the negative association between PIR and severe headache/migraine was more significant in the population of men <60 years of age and those with education greater than or equal to high school.

**Table 5:** Subgroup analysis of the association between PIR and migraine(stratification analysis).

Subgroup	OR (95% CI)	*p*- value
Age(years)		
Age < 45	0.81 (0.76, 0.85)	<0.0001
Age45-60	0.86 (0.79, 0.93)	<0.0001
Age ≥ 60	0.93 (0.84, 1.02)	0.1373
Gender		
Male	0.84 (0.79, 0.90)	<0.0001
Female	0.87 (0.83, 0.92)	<0.0001
Education		
<High School	0.92 (0.85, 1.00)	0.0550
High School	0.87 (0.81, 0.95)	0.0010
>High School	0.82 (0.78, 0.87)	<0.0001

## 4 Discussion

In this study, we explored the possible association between PIR and severe headache/migraine among adults in the United States who were 20 years of age or older. Notably, a negative association was found between PIR and severe headache/migraine [OR = 0.86 95% CI (0.83,0.90)]. Moreover, this correlation was more pronounced in the male population aged <60 and with at least a high school education.

Burch et al. discovered variations in the occurrence of migraine according to employment status and income after analyzing and summarizing many American government health surveys; compared to those who worked part-time (15.6%), those who had no job or had never worked (16.6%), and those who were jobless but had worked (21.4%), full-time workers reported the fewest severe headaches or migraines (13.2%). The highest incidences of migraine were found in individuals with yearly family incomes of less than $35,000 (19.9%), especially those below the poverty line (21.7%) (23). Moreover, a cross-sectional analysis demonstrated a strong negative association between family income and chronic migraine. When the household income level was higher, the prevalence of chronic migraine was lower (24). Compared to those with private insurance (15.1%), those with income below the poverty line (17.1%) and those using Medicaid (26%) had the highest incidence of migraine headaches (25). Similarly, in a longitudinal research of adolescents, poorer family economic status (defined as “below average” or “poverty”) was associated with a ≥ 2-fold increased risk of chronic migraine (26). Therefore, these findings agree with the findings of this investigation.

The triggers that cause migraines differ from person to person. Common triggers include irregular diet, menstruation, stress, overexertion, bright light stimulation, noise, neck discomfort, oversleeping, lack of sleep, weather changes, and specific smells. Typical food triggers include chocolate, soft cheeses, red wine, artificial sweeteners, and monosodium glutamate (MSG) (27–30). Notably, migraine sufferers can reduce the frequency of attacks and alleviate their pain by making lifestyle changes or adjustments to their daily habits (31, 32). Headaches can also be avoided with regular cardiovascular exercise and good sleeping practices (33, 34). Consequently, low-income populations are at higher risk for severe headache/migraine. This may be due to inadequate medical resources. There are also fewer headache specialists in poor rural areas (35). Thus, low-income or uninsured migraineurs are less likely to receive acute-phase treatment (36). The theory assumes that inequality in socioeconomic status could be linked to a different onset of disease, in which individuals with higher household incomes can react to disease more rapidly and efficiently in the early stages. In contrast, those with lower incomes may be unable to respond correctly due to cognitive deficits (37). Prior research has also observed that the prevalence of headache sufferers varies according to socioeconomic status; solutions to improve disparities in headache care include increased health literacy and increased education and training of primary care physicians (38).

Two hypotheses (39, 40) have been proposed to explain the inverse relationship between socioeconomic status and migraine incidence: social selection and social causation. The social selection hypothesis suggests that migraineurs may not be able to fulfill their regular educational and occupational responsibilities. As such, this leads to a decline in social status. In contrast, social causation indicates that those with lower socioeconomic status are more stressed, leading to an increased incidence of migraine. Furthermore, Walter F. et al. found a higher prevalence of migraine among those with lower household incomes (39).

According to a Canadian study (41), stress is a significant contributor to psychological distress, and those with low incomes are more prone to feel it. Moreover, stress is the trigger for nearly 75% of migraine attacks (42). The reason why the negative correlation between PIR and severe headache/migraine was more significant in the population of men <60 years of age and those with ≥high school education may be related to the fact that these populations are more likely to be stressed. Some studies have shown that subjective anxiety symptoms seem to be more severe in male migraineurs than in women (43). Men are more susceptible to work-related stress than women (42, 44, 45). The negative correlation between PIR and severe headache/migraine is more pronounced in men, and this may be associated with more triggers. However, this needs to be confirmed by more studies.

Additionally, this study has some limitations. First, because it was cross-sectional, it was not feasible to establish a causal link between PIR and severe headaches or migraines. Second, a lack of data made it impossible to gather all covariables and adequately categorize headaches. However, our study has some strengths because it is based on conclusions from the NHANES database. All participants were randomly selected through a statistical process using American Census information. Thus, it is representative of the population.

## 5 Conclusion

According to our findings, there is a negative link between PIR and the incidence of severe headaches and migraine. As such, this clarifies how people with severe headaches and migraine should be treated. To decrease the frequency of headaches, it may also be essential to raise the family income level of the patients, support the medical resources available to low-income populations for severe headache/migraine patients, increase scientific awareness, and lessen the triggers of headaches in their daily lives. Additional prospective research is required to validate this discovery.

## Data Availability

The raw data supporting the conclusions of this article will be made available by the authors, without undue reservation.
