# Correction: Association between family income to poverty ratio and severe headache/migraine in the American adults: data from NHANES 1999–2004

**DOI:** 10.3389/fneur.2026.1982659

**Published:** 2026-10-23

**Authors:** Lingling Sun, Rongjian Zhao, Xuemei You, Junpeng Meng, Lin Meng, Haili Di

**Affiliations:** Department of Neurology, Northwest University First Hospital, Xi'an, China

**Keywords:** severe headache/migraine, family income to poverty ratio, NHANES, cross-sectional study, adult

There was a mistake in Table 1 as published. The dietary caffeine intake is right-skewed, the median (interquartile range) is now reported instead of the mean ± SD: 73.00 (4.00, 192.00), 112.00 (26.00, 246.02), and 146.77 (49.70, 289.00) in the low-, middle-, and high-income groups, respectively. In addition, two values in the “Marital status” rows of the high-income group were transcribed incorrectly: “Separated” should read 40 (1.52%) rather than 296 (11.22%), and “Living with partner” should read 100 (3.79%) rather than 277 (5.80%). The corrected column sums to the stated group total of *n* = 2,637.

**Table 1:** Basic characteristics of participants.

Variables	Low income (PIR < 1 *n* = 1,385)	Middle income (1 ≤ PIR < 4 *n* = 4,778)	High income (PIR ≥4 *n* = 2,637)	*P*-value
Age (years)	47.25 ± 18.69	51.12 ± 19.08	50.16 ± 15.71	< 0.001
Gender, *n* (%)				0.361
Male	740 (53.43%)	2,622 (54.88%)	1,471 (55.78%)	
Female	645 (46.57%)	2,156 (45.12%)	1,166 (44.22%)	
Race, *n* (%)				< 0.001
Mexican American	463 (33.43%)	1,100 (23.02%)	281 (10.66%)	
Other Hispanic	90 (6.50%)	229 (4.79%)	49 (1.86%)	
Non-Hispanic White	435 (31.41%)	2,451 (51.30%)	1,888 (71.60%)	
Non-Hispanic Black	347 (25.05%)	882 (18.46%)	332 (12.59%)	
Other Race	50 (3.61%)	116 (2.43%)	87 (3.30%)	
Education, *n* (%)				< 0.001
< High school	787 (56.82%)	1,560 (32.65%)	211 (8.00%)	
High school	268 (19.35%)	1,332 (27.88%)	500 (18.96%)	
>High school	330 (23.83%)	1,886 (39.47%)	1,926 (73.04%)	
Marital status, *n* (%)				< 0.001
Married	545 (39.35%)	2,636 (55.17%)	1,881 (71.33%)	
Widowed	130 (9.39%)	506 (10.59%)	125 (4.74%)	
Divorced	185 (13.36%)	483 (10.11%)	195 (7.39%)	
Separated	99 (7.15%)	141 (2.95%)	40 (1.52%)	
Never married	297 (21.44%)	735 (15.38%)	296 (11.22%)	
Living with partner	129 (9.31%)	277 (5.80%)	100 (3.79%)	
Diabetes, *n* (%)				< 0.001
Yes	176 (12.71%)	498 (10.42%)	155 (5.88%)	
No	1,194 (86.21%)	4,203 (87.97%)	2,455 (93.10%)	
Borderline	15 (1.08%)	77 (1.61%)	27 (1.02%)	
Hypertension, *n* (%)				0.011
Yes	433 (31.26%)	1,590 (33.28%)	790 (29.96%)	
No	952 (68.74%)	3,188 (66.72%)	1,847 (70.04%)	
Stroke, *n* (%)				< 0.001
Yes	57 (4.12%)	169 (3.54%)	50 (1.90%)	
No	1,328 (95.88%)	4,609 (96.46%)	2,587 (98.10%)	
Coronary heart disease, *n* (%)				0.782
Yes	62 (4.48%)	232 (4.86%)	131 (4.97%)	
No	1323 (95.52%)	4,546 (95.14%)	2,506 (95.03%)	
Smoke status, *n* (%)				< 0.001
Yes	891 (64.33%)	2,710 (56.72%)	1,290 (48.92%)	
No	494 (35.67%)	2068 (43.28%)	1,347 (51.08%)	
Drink status, *n* (%)				< 0.001
Yes	320 (23.10%)	902 (18.88%)	295 (11.19%)	
No	1,065 (76.90%)	3,876 (81.12%)	2,342 (88.81%)	
BMI(kg/m^2^)	32.70 ± 8.11	32.72 ± 7.94	32.40 ± 8.20	0.135
Caffeine intake (mg/d)	73.00 (4.00, 192.00)	112.00 (26.00, 246.02)	146.77 (49.70, 289.00)	
Migraines, *n* (%)				< 0.001
Yes	376 (27.15%)	970 (20.30%)	368 (13.96%)	
No	1,009 (72.85%)	3,808 (79.70%)	2,269 (86.04%)	

Reviewing the original statistical results of this study, the PIR groups were as follows: PIR < 1; 1 ≤ PIR < 4; PIR ≥ 4. All statistical processes were conducted under these groups. Therefore, the group description in Table 1 is incorrect. The corrected Table 1 appears below:

In the published article, there was an error in the **Methods** section, “*2.3 Statistical analysis*”. The sentence describing the tests used to compare participant characteristics across the three PIR groups does not describe the analysis that was actually performed. All baseline comparisons reported in Table 1 were generated in EmpowerStats (version 2.0) and R (version 4.1.3): continuous variables were compared using the Kruskal–Wallis rank-sum test and categorical variables using the chi-square test. No comparison in Table 1 was performed using a *t*-test, and none of the *p*-values reported in Table 1 have changed. Additionally, there is an error in the PIR grouping description.

A correction has been made to **Methods**, section 2.3, paragraph 1. This sentence previously stated: “T-test and chi-square test were used to characterize participants' demographic information after PIR grouping (PIR < 1, PIR1-4, PIR > 4) in the study.”

The corrected sentence appears below:

“Continuous variables are presented as mean ± SD and were compared across the three PIR groups (PIR < 1, 1 ≤ P IR < 4, PIR ≥ 4) using the Kruskal–Wallis rank-sum test; categorical variables are presented as n (%) and were compared using the chi-square test.”

According to the original statistical results of this study, the PIR groups were as follows: PIR < 1, 1 ≤ PIR < 4, PIR ≥ 4; All statistical processes were conducted within the above groups. Therefore, there are still multiple errors in the PIR group descriptions in the original article, as follows:

In the “Abstract; **Results**” section:

The corrected sentence appears below: The variable PIR was categorized as the low-income (PIR < 1), the middle-income (1 ≤ PIR < 4), and the high-income (PIR ≥ 4). Notably, there was a significant difference in trend for the high-income group (PIR ≥ 4) compared to the control low-income group (PIR < 1) (all P for interaction < 0.05).

In the “*2.2 Study variables*” section:

The corrected sentence appears below: We then categorized these into three classes: the low-income (PIR < 1), the middle-income (1 ≤ PIR < 4), and the high-income (PIR ≥ 4).

In the “*3.1 Baseline characteristics of participants*” section:

The corrected sentence appears below: There were 1,385 (15.74%) participants with low-income (PIR < 1), 4,778 (54.30%) with middle-income (1 ≤ PIR < 4), and 2,637 (29.96%) with high-income (PIR ≥ 4). Notably, the low-income individuals (PIR < 1) were younger, more likely to have less than a high school education, had lower coffee intake, and had a higher prevalence of severe headaches/migraines compared to the high-income individuals (PIR ≥ 4).

In the “*3.3 Correlation of PIR with severe headache/migraine*” section:

The corrected sentence appears below: After triple categorizing the variable PIR (PIR < 1, 1 ≤ PIR < 4, PIR ≥ 4), there was a significant difference in trend for the high-income group (PIR ≥ 4) compared to the low-income group (PIR < 1).

In the “Table 3” section

The corrected Table 3 is as follows:

**Table 3:** The association between family PIR and migraine.

Exposure	Model I OR(95%CI) P	Model II OR(95%CI) P	Model III OR(95%CI) *P*
PIR	0.83 (0.80, 0.86) < 0.0001	0.85 (0.82, 0.89) < 0.0001	0.86 (0.83, 0.90) < 0.0001
PIR classification			
Low income (PIR < 1)	Reference	Reference	Reference
Middle income (1 ≤ PIR < 4)	0.68 (0.60, 0.78) < 0.0001	0.78 (0.68, 0.91) < 0.0001	0.80 (0.69, 0.92) 0.0028
High income (PIR ≥ 4)	0.44 (0.37, 0.51) < 0.0001	0.52 (0.43, 0.63) < 0.0001	0.54 (0.45, 0.65) < 0.0001
*P* for trend	< 0.001	< 0.001	< 0.001

Model I: None covariates were adjusted.

Model II: Age, race, marital status, education and coronary heart disease were adjusted.

Model III: Age, race, marital status, education, coronary heart disease, drink and smoke status, Caffeine intake, Hypertension, diabetes, stroke, and BMI (body mass index) were adjusted.

PIR is the ratio of family income to poverty.

The original version of this article has been updated.

